# Simple Theoretical Results on Reversible Fouling in Cross-Flow Membrane Filtration

**DOI:** 10.3390/membranes9040048

**Published:** 2019-04-03

**Authors:** Pierre Haldenwang, Braulio Bernales, Pierrette Guichardon, Nelson Ibaseta

**Affiliations:** Aix Marseille Univ, CNRS, Centrale Marseille, M2P2, 38 rue Joliot-Curie, 13451 Marseilles, France; pierre.haldenwang@univ-amu.fr (P.H.); braulio.bernales@gmail.com (B.B.); nelson.ibaseta@ec-marseille.fr (N.I.)

**Keywords:** membrane separation, cross-flow filtration, polarization of concentration, limiting flux, reversible fouling, Starling–Darcy boundary conditions

## Abstract

In cross-flow membrane filtration, fouling results from material deposit which clogs the membrane inner surface. This hinders filtration, which experiences the so-called limiting flux. Among the models proposed by the literature, we retain a simple one: a steady-state reversible fouling is modelled with the use of a single additional parameter, i.e., $N_{d}$, the ratio of the critical concentration for deposition to the feed concentration at inlet. To focus on fouling, viscous pressure drop and osmotic (counter-)pressure have been chosen low. It results in a minimal model of fouling. Solved thoroughly with the numerical means appropriate to enforce the nonlinear coupling between permeation and concentration polarization, the model delivers novel information. It first shows that permeation is utterly governed by solute transfer, the relevant non-dimensional quantities being hence limited to $N_{d}$ and $Pe_{in}$, the transverse Péclet number. Furthermore, when the role played by $N_{d}$ and moderate $Pe_{in}$ (say $Pe_{in}<40$) is investigated, all results can be interpreted with the use of a single non-dimensional parameter, $F_{l}$, the so-called fouling number, which simply reads $F_{l}\equiv Pe_{in}N_{d}^{-1}$. Now rendered possible, the overall fit of the numerical data allows us to put forward analytical final expressions, which involve all the physical parameters and allow us to retrieve the experimental trends.

## 1. Introduction

Membrane filtration systems are conceived to perform species separation. They consist of selecting semi-permeable membranes that retain the targeted species, while some others cross the membranes. The retained species then accumulate in a mass boundary layer that develops along the membrane inner surface, giving rise to the so-called polarization of concentration. Such an increase in concentration at the membrane, which results from the competition between advection towards the membrane and diffusion back to the bulk, may induce two types of hindrance to permeation: osmotic (counter-)effects and membrane fouling. This is why particular efforts have been paid to reduce the polarization of concentration through special devices as obstacles or vortices that can prevent the development of a mass boundary layer over a long distance.

On the one hand, the polarization of concentration increases the osmotic pressure between both sides of the membrane. This can result in a severe reduction of the effective operating pressure. This effect, typical of reverse osmosis and nanofiltration, has been the object of a previous theoretical study [1], the result of which showed that osmosis causes an inflection of logarithmic type in permeation versus pressure [1]. In the present article, which is our first contribution on fouling, we choose parameters that minimize the effects of osmotic (counter-)pressure. The focus on fouling may help to discriminate fouling from osmotic effects—which are often intertwined by pointing out a behaviour specific to fouling. In this way, the present paper will confirm that limiting flux is caused by fouling, while logarithmic growth is due to osmosis.

On the other hand, membrane fouling is known to affect most of the filtration devices. In particular, fouling corresponds to the most dangerous hindrance that can affect ultrafiltration (although some analyses showed that fouling and osmotic effects may, however, appear as deeply interconnected [2]). It consists of material deposition due to various possible phenomena: the solution precipitates, a gel develops on the membrane, the over-saturation induces the growth of a solid layer along the membrane, the solute is substantially adsorbed within the membrane pores, etc. This additional material results in an increase in membrane resistance leading to the limiting flux and, finally, in operational cost due to a larger energy demand, an additional effort for cleaning and a shorter membrane life.

In the experimental practice, two kinds of fouling are mainly considered. Fouling is conceived as reversible if the membrane properties are recovered after its cleaning by the solvent, while it is said irreversible when fouling remains after cleaning. Numerous investigations have been devoted to point out and characterize the nature of the fouling. An important literature endeavours to describe the plausible mechanisms and their principal factors, as well as supplying elements for modelling. Of particular interest, the critical flux concept has been established in the mid 1990s [3,4,5] to describe the flux below for which fouling remains insignificant. Further developments on critical flux which concern theory, experimental measurements and applications are summarised in the review paper [6]. Worthy of note is the experimental method that permits the differentiation between reversible and irreversible fouling [7]. It is evidently desirable to characterize the spatial dependence of fouling. Thus, experimentalists have carried out time-dependent local measurements of fouling occurrence and growth rate, which have resorted to various methods: X-ray techniques [8], optical investigation techniques [9], or nuclear magnetic resonance [10].

Because solute is carried towards the membrane inner surface by the flow, the understanding of the concentration polarization phenomenon requires to characterize the hydrodynamics related to membrane cross-flow filtration. In that domain, the art started early with the seminal papers [11,12,13,14] which supposed that solute transport did not affect the flow. The current state in analytical theories [15,16,17,18,19] focus on the possible couplings between filtration and pressure variation along the inner side of the membrane.

On the other side, for given particular filtration flows, concentration polarization has benefited from numerous theoretical investigations. The most popular flows are the transverse or quasi-transverse flows, which sustain the various theories linked to the “1D film model”. Among the earliest contributions is the popular “gel layer” polarization model [20]. Further analytical contributions have extended the earliest approaches to more realistic concentration boundary layers [21,22,23,24,25,26,27,28,29,30]. Note that these models suppose the existence of a thin film wherein polarization occurs, the axis-evolution of this film being potentially envisaged in the most recent ones.

Although the general coupled problem seems out of the reach of the current analytical methods, it has however been possible to find particular operating domains capable of reducing the strength of the coupling between hydrodynamics and solute transfer, and leading the theoretical approach to analytical expressions. This is the case of the “HP-LR limit” (for High Pressure and Low Recovery), which allowed us to derive an exact expression for the overall solute transport coupled with its related Berman flow [1]. This solution clearly exhibited the phenomenon of concentration polarization, which was combined with osmotic (counter-)effects to predict the actual permeation. In its domain of validity, such an exact approach moreover exhibits the so-called “inflected flux” phenomenon (i.e., some logarithmic behaviour observed in the experiments as operating pressure increases). Note additionally that certain analytical studies searched after similarity solutions (see, e.g., [31,32]).

To end with the theoretical approach, the numerical approach has largely been used to model fouling. Facing the difficulties for the analytical approach to cope with the overall coupling between mass transport and flow, one usually resorts to numerics. In particular, the numerical approach is seemingly indispensable for taking the solute axial variations into account [33]. As a result, numerous numerical models consider a solute boundary layer growing along the channel [34,35,36,37,38,39,40]. These numerical models generally solve the Navier–Stokes equations. However, when no stiff variations are present, it can be demonstrated that the framework of the Prandtl parabolic approximation is valid for modelling standard membrane filtration in the cross-flow configuration. As a result, Prandtl equations offer a simplified model which allows us to reduce the computational cost and to easily enforce the nonlinear coupling between filtration and concentration polarization at the membrane surface [41].

**Figure 1:**
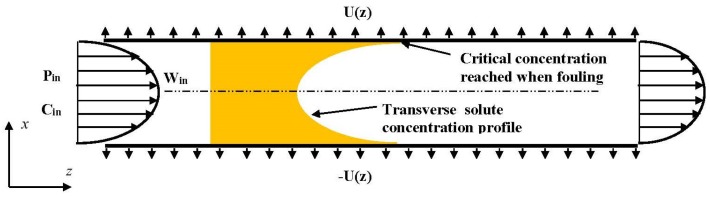
Sketch of the separation system: feed flow conditions in pressure, velocity and concentration profiles are imposed at the channel entrance. When fouling arises, concentration $C_{d}$ is maintained at the walls.

In this way, the present paper numerically investigates the reversible fouling within the framework of a 2D channel flow. Two semi-permeable parallel walls compose the 2D channel as schematically shown in Figure 1. The channel is of length L and spacing 2d. To reduce the computational cost, and thus to conduct a nearly exhaustive study of all the relevant parameters, the steady conservation laws (continuity, Navier–Stokes and solute convective-diffusive equations) are considered within the framework of the Prandtl approximation [41]: pressure only depends on the axial coordinate (z) and all phenomena of diffusion along the axis are neglected. For the sake of simplifying the discussion, the present work considers only one species, whereas the membrane selectivity is perfect (the species is fully rejected). Furthermore, as soon as the polarization of concentration reaches a critical value, called concentration of deposit and hereinafter denoted $C_{d}$, any additional solute molecule immediately settles on the membrane inner surface, or in the membrane texture itself. In other words, $C_{d}$ is the concentration of equilibrium between solution and deposit. This is therefore the maximum concentration that can be met in the solution. Such an assumption, which considers the kinetics of deposit and dissolution as infinitely fast, is generally admitted for a large set of membrane filtration systems (see, for instance, [42,43]). Lastly, to focus on the features specifically related to fouling, pressure drop and osmotic (counter-)effects are supposed very low.

The article is organized as follows. Section 2 presents the physical model and explains the role of the independent parameters. Section 3 exposes the mathematical model and discusses the basic assumptions. In Section 4, the numerical method is described focusing on the specific iterative procedure when fouling occurs. Section 5 is devoted to the numerical results, their classification with respect to the fouling number $F_{l}$ as defined below. The fit with an analytical expression, and the final expression of all the results is investigated in Section 6. Section 7 deals with the dimensional reconstruction of the permeate flux. The role played by the main physical parameters is investigated. Conclusions and perspectives concern Section 8.

## 2. The Physical Model

### 2.1. Model of Reversible Fouling

The selected model of fouling implies that the concentration in the solution varies in the range limited by $C_{in}$, the initial concentration, and $C_{d}$, the concentration of coexistence with the deposit. The model introduces the non-dimensional number $N_{d}$, the so-called deposit number, which is the ratio of $C_{d}$ to feed concentration $C_{in}$. In the article, *C*, the non-dimensional concentration (in units of $C_{in}$) will therefore vary between 1 and $N_{d}$. According to its principles, the present numerical approach does not imply any restriction on the value of $N_{d}$. However, since Section 6 is based on a stage of analytical fit that will lose its meaning when $N_{d}\leq 2$, we hereinafter suppose $N_{d}>2$.

In the absence of fouling, i.e., for $1\leq C<N_{d}$ at the membrane surface, the steady state implies that an equilibrium between species mass transfers by filtration velocity and by mass diffusion back to the bulk is reached. In other words, a Robin boundary condition on concentration (see below) is imposed at the membrane surface, as long as $C<N_{d}$. When fouling arises, another steady situation appears. We suppose a fast kinetics of deposition, in such a manner that the concentration cannot be larger than $N_{d}$. A certain wall resistance to filtration occurs due to material deposit. The layer of material deposit reaches a steady state when the filtration is such that $C=N_{d}$, which indicates that the deposit layer is in equilibrium with the solution at the membrane surface. In other words, the Dirichlet boundary condition $C=N_{d}$ is imposed on the concentration at the membrane surface. The filtration will therefore be a by-product of the latter condition, since the solute diffusion back to the bulk is still balanced by the solute transport towards the membrane. The filtration resistance due to the deposit layer will eventually be deduced from the overall approach. It is worthy of note that the above assumptions are characteristic of reversible fouling since no coexistence is envisaged between material deposit and solution with $C<N_{d}$.

To simplify the analysis, we postulate that the solute rejection is total. This assumption allows us to suppress a parameter characterizing the transport in the membrane and does not correspond to a severe restriction in numerous situations [44]. Even though the latter assumption simplifies the situation for the solute, modelling the strength of the solvent flow that crosses the permeable walls is not a trivial task, since it depends on various physical effects. We first have in mind the pressure difference between both sides of the wall (i.e., the operating pressure $P_{in}$), which can vary along the channel, due to viscous effects. In the separation of electrolytes or various organic molecules, osmosis (which reduces the net operating pressure) is often expected to cause the dominant limitation in filtration. As we want to focus the present study on fouling only, we here suppose that the osmotic (counter-)pressure is low in comparison with the operating pressure. To sum up, the present analysis specifically targets the polarization of concentration and the possible additional resistance to filtration that can occur in the context of a reversible deposit of material.

### 2.2. The Control Parameters

For the sake of establishing the simplest physical concepts, all properties relative to the solution and the membrane are supposed uniform (uniformity is specified by the subscript “0” in the labelling). We thus denote by $I_{0}$ the uniform membrane resistance to filtration without fouling [$I_{0}^{-1}$ being the membrane permeability to solvent]. As for the subscript “in”, it is devoted to quantities that correspond to conditions of injection, i.e., experimentally considered as control parameters. Hence, the “clean water” filtration velocity at entrance is given by

(1) $$U_{in}\equiv \frac{P_{in}}{I_{0}}.$$

The “clean water” concept corresponds to a feed flow of pure solvent. $U_{in}$ is hence an upper bound of the permeation velocity; it also gives a scale of reference for the transverse velocity. Then, $R_{in}$, the transverse Reynolds number of (clean water) permeation at entrance is a parameter that characterizes the channel flow. It reads

(2) $$R_{in}\equiv \frac{\rho_{0}P_{in}d}{\mu_{0}I_{0}},$$

where $\rho_{0}$ is the fluid density, $\mu_{0}$ is the fluid dynamic viscosity and *d*, the channel half-spacing). In standard filtration system, $R_{in}$ compares transverse inertial with viscous forces, and generally belongs to the range $\left[10^{-3},10^{-1}\right]$. In certain systems of microfiltration, $R_{in}$ can approach O(1), a situation that can produce some dramatic change in permeation even in case of pure solvent filtration [16]. $Pe_{in}$, the Péclet number of “clean water” permeation at entrance, is defined by

(3) $$Pe_{in}\equiv ScR_{in}=\frac{P_{in}d}{D_{0}I_{0}},$$

where Sc, the Schmidt number, is defined as $Sc=\mu_{0}{\left(D_{0}\rho_{0}\right)}^{-1}$, with $D_{0}$, the (uniform) solute diffusivity in the feed (In Section 8, it will be suggested that the present study might be extended to turbulent flows by substituting $D_{turb}$ for $D_{0}$]. $Pe_{in}$ compares transverse advection flux with back diffusion flux, and can be interpreted as a dimensionless operating pressure, that can be easily varied in the experiments. We are fully aware that our simplifying assumptions of uniform $\rho_{0}$, $\mu_{0}$ and $D_{0}$ present a certain level of weakness in the case of high polarization (i.e., $N_{d}\gg 1$).

Let us identify the independent parameters; the experimentalists consider as easily adjustable $P_{in}$, the overall pressure at channel entrance, $W_{in}$, the inlet axial mean velocity, and $C_{in}$. Considering the part played by $W_{in}$, we expect three main contributions: (a) axial velocity determines inlet solvent flow rate and solute mass rate; (b) flow rate induces viscous pressure drop; and (c) axial velocity controls the solute boundary layer establishment.

To help the discussion on effect (a), we define $L_{de}$, the so-called dead-end length, which is the exhaustion length in the case of “clean water” flow (and a lower bound for the actual depletion length in a real case of filtration):

(4) $$L_{de}\equiv \frac{I_{0}W_{in}d}{P_{in}}.$$

$L_{de}$ provides us with a length of reference for the longitudinal variations. Therefore, *L*, the dimensional channel length will be reduced with the use of $L_{de}$ through the definition of λ, the non-dimensional channel length, written as follows:

(5) $$\lambda \equiv \frac{L}{L_{de}}=\frac{P_{in}L}{W_{in}I_{0}d}.$$

To focus on mass transfer, and since the effects of pressure change are quite well understood [13,15,16], we shall only consider the situations where effect (b) (i.e., pressure axial change) is negligible. When the transverse Reynolds number is small (i.e., $R_{in}\ll 1$), it is known from Berman theory that the pressure variations are negligible along the channel, if the Hagen–Poiseuille pressure drop $\Delta P_{HP}$ is negligible too. More precisely, $\Delta P_{HP}/P_{in}$ reads

(6) $$\frac{\Delta P_{HP}}{P_{in}}=3\frac{\mu_{0}W_{in}L}{d^{2}P_{in}}=3\lambda \frac{\mu_{0}W_{in}L_{de}}{d^{2}P_{in}}=3\lambda \alpha^{2},\;\mathrm{with}\;\alpha^{2}=\frac{\mu_{0}W_{in}^{2}I_{0}}{P_{in}^{2}d}.$$

Here, α is a non-dimensional quantity that appears in the Regirer theory, which provides us with the basic solution that accounts for the pressure drop effect on permeation [13,15]. In the standard ultrafiltration (UF) processes, $\Delta P_{HP}$ can be estimated to about $\Delta P_{HP}=10$ kPa, to be compared with an operating pressure of several hundreds of kPa, the operating pressure in UF. To neglect the pressure drop effects hereinafter, we shall suppose $3\lambda \alpha^{2}\ll 1$. As for effect (c), which concerns the establishment of the concentration boundary layer, the present (laminar) study will show that effect (c) is already accounted for in the treatment of effect (a). In other words, modifying $W_{in}$ only changes λ, the actual non-dimensional channel length (and α, a number that has already been chosen to be small).

Lastly, $C_{in}$, the third easily adjustable parameter, determines the strength of the osmotic effects in the absence of polarization. In a general manner, the order of magnitude of $P_{in}^{osm}$, the osmotic pressure, more or less follows the van’t Hoff law, which reads in the absence of polarization

(7) $$P_{in}^{osm}=iRTC_{in},$$

where *i* is the number of dissociated entities (ionic or neutral) per solute molecule in the solution. *R* and *T* are respectively the perfect gas constant and temperature. Our purpose is to neglect those effects. We are then invited to compare $P_{in}^{osm}$ with $P_{in}$, and this leads us to introduce $N^{osm}$, the so-called osmotic number [1] as

(8) $$N^{osm}\equiv \frac{iRTC_{in}}{P_{in}}.$$

Hereinafter, we now postulate $N^{osm}\ll 1$. This assumption allows us to consider the osmotic effects as feeble (but not zero to keep a marker of the polarization). Now, we can describe the role of the control parameters that are easily adjusted by the experimentalists on the basic dimensionless parameters. Hereafter, $Pe_{in}$ is the dimensionless form of the trans-membrane pressure (TMP), $N_{d}^{-1}$ the dimensionless form of the feed concentration, while $\lambda^{-1}$ is proportional to $W_{in}$ for fixed TMP.

### 2.3. Only Three Relevant Numbers

This paragraph is devoted to present the dimensional analysis of the mass transfer system that we are faced with. Gathering all the physical parameters that have been introduced above, we have to consider eleven independent parameters, namely

(9) $$\{C_{in},C_{d},D_{0},\left(iRT\right),d,L,W_{in},\rho_{0},\mu_{0},P_{in},I_{0}\}.$$

Classical Dimensional Analysis predicts that only seven numbers should rule flow and mass transfer. Let us again consider the six numbers quoted already, plus a new one denoted by β

(10) $$N_{d}=\frac{C_{d}}{C_{in}};\;Pe_{in}=\frac{P_{in}d}{D_{0}I_{0}};\;\lambda =\frac{P_{in}L}{W_{in}I_{0}d},$$

(11) $$R_{in}=\frac{\rho_{0}P_{in}d}{\mu_{0}I_{0}};\;\alpha =\frac{W_{in}}{P_{in}}\sqrt{\frac{\mu_{0}I_{0}}{d}};\;N_{osm}=\frac{iRTC_{in}}{P_{in}};\;\beta =\frac{\mu_{0}}{I_{0}d}.$$

The order of magnitude of the last number β has been analysed in [15]. Actually, β compares the transverse pressure drop with TMP (or compares the effective sectional area of a membrane porous with the product of channel width and effective membrane thickness). As a result, β varies in the range $\left[10^{-8},10^{-3}\right]$ and may be considered as vanishing in most situations of filtration. Consequently, the last three numbers $\left\{\alpha ,N_{osm},\beta \right\}$ may leave the framework of our study on fouling. As often observed in steady phenomena controlled by mass diffusion, our numerical experiments conducted in Section 5 will show that $R_{in}$ also plays a feeble role in the present problem of mass transfer. In other words, $\left\{Pe_{in},N_{d},\lambda \right\}$ (i.e., the set given by Equations (10)) are the only three relevant parameters of the present study on fouling. Hence, a nearly-exhaustive numerical study can be carried out, if a fast computation of the basic conservation laws is achieved. To our knowledge, no contribution to date exists that reduces the study on fouling to a three-parameter problem that can be solved comprehensively. The latter point is the purpose of the next section.

## 3. The Prandtl System

In a previous contribution [41], it has been demonstrated that the Prandtl approximation may apply in the standard configurations of filtration. An incompressible Newtonian fluid flow of velocity $\left\{\tilde{U},\tilde{W}\right\}$, pressure $\tilde{P}$ and concentration $\tilde{C}$, is considered within the open domain $]-d<\tilde{x}<d\left[\;\times \;\right]0<\tilde{z}<L[$. To write the Prandtl system in non-dimensional form, we define the following set of non-dimensional unknowns and variables:

(12) $$\left\{u,v\right\}\equiv \left\{\frac{\tilde{U}}{U_{in}},\frac{\tilde{W}}{W_{in}}\right\},\;p\equiv \frac{\tilde{P}}{P_{in}},\;C\equiv \frac{\tilde{C}}{C_{in}},\;x\equiv \frac{\tilde{x}}{d},\;z\equiv \frac{\tilde{z}}{L_{de}},$$

where the superscript ($\tilde{}$) distinguishes the dimensional form of the concerned unknowns. In this way, the computational domain becomes ]−1<x<1[×]0<z<λ[.

The derivation of the Prandtl set of equations uses the following arguments [41]. As long as feed axial velocity is large in comparison with permeation velocity, the transverse variation of pressure can be neglected in all filtration systems. Pressure is hence reduced to a single-variable quantity, a function of the axial position *z*, only. In the same vein, all axial diffusion terms can be cancelled in the conservation laws, except when a mathematical discontinuity happens (as discussed later). The resulting set of equations then reads

(13) $$\begin{array}{ccc} \frac{\partial u}{\partial x}+\frac{\partial w}{\partial z} & = & 0, \end{array}$$

(14) $$\begin{array}{ccc} \frac{\partial p}{\partial x} & = & 0, \end{array}$$

(15) $$\begin{array}{ccc} -\frac{1}{\alpha^{2}}\frac{\partial p}{\partial z}+\frac{\partial^{2}w}{\partial x^{2}} & = & R_{in}\left(u\frac{\partial w}{\partial x}+w\frac{\partial w}{\partial z}\right), \end{array}$$

(16) $$\begin{array}{ccc} \frac{\partial^{2}C}{\partial x^{2}} & = & Pe_{in}\left(u\frac{\partial C}{\partial x}+w\frac{\partial C}{\partial z}\right), \end{array}$$

together with appropriate axial boundary conditions and with the following transverse boundary conditions at the wall (x=±1, ∀z):

(17) $$\begin{array}{ccc} w & = & 0\;\;\;\;,\;\;\;\;u\;C=\frac{1}{Pe_{in}}\frac{\partial C}{\partial x}, \end{array}$$

(18) $$\begin{array}{ccc} u & = & p-N_{osm}C\;,\;\;\;\mathrm{in}\;\mathrm{the}\;\mathrm{absence}\;\mathrm{of}\;\mathrm{fouling}, \end{array}$$

(19) $$\begin{array}{ccc} C & = & N_{d}\;\;,\;\;\;\;\mathrm{in}\;\mathrm{presence}\;\mathrm{of}\;\mathrm{fouling}. \end{array}$$

Note that boundary conditions (18) are often referred to as Starling–Darcy boundary conditions. The above differential system calls for the following remarks.

**Remark** **1.** *Equations (15) and (16) are clearly of a parabolic type, the pressure being in every section the Lagrange multiplier that permits constraint (13). The forthcoming numerical method will exploit these mathematical features. Furthermore, a parabolic system admits computational methods of “time marching” type, which only requires “initial” conditions at inlet. Hence, “the appropriate boundary conditions” mentioned above are reduced to entrance conditions. In other words, suitable entrance profiles on C and w are only required at z=0.*

**Remark** **2.** *If the above inlet data at z=0 are symmetrical, the laminar solution of the system will develop symmetrically with respect to x=0, the line of symmetry. Therefore, assuming symmetrical boundary conditions at x=0 allows us to save half of the computational effort, the computational domain being reduced to ]0<x<1[×]0<z<λ[.*

**Remark** **3.** *Conservation laws are nonlinear by nature. In filtration problems, their nonlinearity is increased by boundary condition (17) at the membrane surface, which nonlinearly combines permeation velocity and solute concentration. This coupling is tremendously important because it governs polarization before the occurrence of fouling. Therefore, this nonlinearity will be iteratively enforced in the numerical scheme, as described below.*

To summarize the present section, Prandtl approximations, constant physical properties, reversible fouling with infinitely fast kinetics and assumption of total solute rejection allow us to construct a minimal model, the validation of which will lie in its capability of predicting the general experimental trends. In terms of computational cost, the Prandtl approximation represents a sizeable simplification. As for the studied range of parameters, this has some price to pay, as will be discussed below.

## 4. Numerical Approach

Since the governing system is now of parabolic type, the numerical solution resorts to the classical implicit methods for solving the systems that are of heat equation type. We hence adopt the terminology generally used for discretizing the heat equation with finite differences, the axial coordinate playing the role of time in the latter equation. Let us define $w_{j}^{n}$ [resp. $u_{j}^{n}$ and $C_{j}^{n}$], the value of the unknown *w* [resp. *u* and *C*] at the node $x_{j}=j\Delta x,z^{n}=n\Delta z$ for 0≤j≤J and 0≤n≤N, where Δx=1/J and Δz=λ/N are the mesh sizes in both directions. The “time marching” supposes that all transverse profiles are known up to section $z^{n}$. The purpose of the numerical scheme is to compute all profiles in section $z^{n+1}$.

### 4.1. “Time Marching” in the Axial Direction

By extrapolation from the previous transverse sections $\{z^{n}$, $z^{n-1}$, $z^{n-2}$, ..., $z^{n-\xi }\}$, where (ξ+1) corresponds to the extrapolation order, it is easy to obtain a guess of these next profiles, denoted ${\hat{w}}_{j}^{n+1}$ (resp. ${\hat{u}}_{j}^{n+1}$ and ${\hat{C}}_{j}^{n+1}$). We afterwards have to choose an implicit discretization for the first derivative with respect to the longitudinal direction, the general form of which reads

(20) $$\frac{\partial w}{\partial z}{|}_{j}^{n+1}\approx \frac{1}{\Delta z}\sum_{\zeta =0}^{\zeta =\xi +1}\sigma_{\zeta }w_{j}^{n+1-\zeta },$$

where (ξ+1) corresponds to the order of the discretization and the set $\left\{\sigma_{\zeta }\right\}$ is composed of suitable coefficients. Differential systems (13)–(16) then become after the discretization process with respect to *z*

(21) $$\begin{array}{ccc} {\hat{w}}^{n+1}\frac{\sigma_{0}}{\Delta z}C^{n+1}-\frac{1}{Pe_{in}}{\frac{\partial^{2}C}{\partial x^{2}}}^{n+1} & = & S^{C}\left(x\right)\equiv \\ {\hat{w}}^{n+1}\sum_{\zeta =1}^{\zeta =\xi +1}\frac{\sigma_{\zeta }}{\Delta z}C^{n+1-\zeta } & - & {\hat{u}}^{n+1}{\frac{\partial \hat{C}}{\partial x}}^{n+1},\;\;\; \end{array}$$

(22) $$\begin{array}{ccc} {\hat{w}}^{n+1}\frac{\sigma_{0}}{\Delta z}w^{n+1}-\frac{1}{R_{in}}{\frac{\partial^{2}w}{\partial x^{2}}}^{n+1}+\frac{1}{R_{in}\alpha^{2}}\frac{\sigma_{0}}{\Delta z}p^{n+1} & = & S^{W}\left(x\right)\equiv \\ -{\hat{w}}^{n+1}\sum_{\zeta =1}^{\zeta =\xi +1}\frac{\sigma_{\zeta }}{\Delta z}w^{n+1-\zeta }-\frac{1}{R_{in}\alpha^{2}}\sum_{\zeta =1}^{\zeta =\xi +1}\frac{\sigma_{\zeta }}{\Delta z}p^{n+1-\zeta } & - & {\hat{u}}^{n+1}{\frac{\partial \hat{w}}{\partial x}}^{n+1}.\;\;\; \end{array}$$

Note that source terms $S^{C}$ and $S^{W}$ are two functions of the transverse coordinate *x*, explicitly known at section $z^{n+1}$. Furthermore, ODE (21) (ODE, for ordinary differential equation) can be identified with the heat equation with source term. It has to be complemented with homogeneous Neumann boundary conditions (i.e., symmetry w.r.t. x=0), and the Robin one (i.e., mass budget $Pe_{in}{\hat{u}}^{n+1}C^{n+1}=\partial C^{n+1}/\partial x$ at x=1). ODE (22) has to be complemented with homogeneous Neumann boundary conditions (i.e., symmetry w.r.t. x=0) and homogeneous Dirichlet ones (i.e., no slip at x=1). Since ODE (22) involves $p^{n+1}$ as an additional unknown in comparison with the heat equation, a complementary constraint must be provided to close the problem. Actually, this additional scalar equation depends on the fact that a critical concentration has been reached, or not.

On the one hand, at a small polarization of concentration, the critical concentration is not attained and no deposit can take place. Therefore, the resistance to permeation is known (i.e., $I_{0}$) and condition (18) holds at the membrane. The latter condition implies $U^{n+1}\equiv u_{J}^{n+1}$, the permeation velocity at the membrane, which can be related to the field of *w* in every section after integration of the incompressibility constraint (13) in the section. This yields the following discretized form of the incompressibility constraint

(23) $$U^{n+1}+\frac{\sigma_{0}}{\Delta z}\sum_{j=0}^{j=J}\chi_{j}w_{j}^{n+1}=\sum_{\zeta =1}^{\zeta =\xi +1}\frac{\sigma_{\zeta }}{\Delta z}\left\{\sum_{j=0}^{j=J}\chi_{j}w_{j}^{n+1-\zeta }\right\}.$$

Equation (23) leads us to derive a discretized form of the Starling–Darcy condition on permeation at a membrane for an incompressible fluid. It couples the concentration at the membrane surface with the axial velocity field as

(24) $$\frac{\sigma_{0}}{\Delta z}\sum_{j=0}^{j=J}\chi_{j}w_{j}^{n+1}+p^{n+1}-N_{osm}C^{n+1}=\sum_{\zeta =1}^{\zeta =\xi +1}\frac{\sigma_{\zeta }}{\Delta z}\left\{\sum_{j=0}^{j=J}\chi_{j}w_{j}^{n+1-\zeta }\right\},$$

where $\left\{\chi_{j},j=0,\ldots ,J\right\}$ is a suitable set of coefficients derived from the classical numerical methods for integration. Note that differential Equation (13) has been reduced to the scalar constraint (24). This is consistent with the number of unknowns and occurs as a counterpart of the fact that pressure, the Lagrange multiplier related to incompressibility constraint, is reduced to scalar $p^{n+1}$ in the whole section.

On the other hand, as polarization of concentration increases, the critical concentration $N_{d}$ is exceeded at some critical section $z_{c}$ and a deposit arises until the resulting additional resistance reduces the permeation in such a manner that the concentration polarization becomes in equilibrium with the deposit. In other words, in case of (steady) fouling, the missing condition is

(25) $$C_{J}^{n+1}\equiv C\left(x=1,z^{n+1}\right)=N_{d}$$

in non-dimensional form.

### 4.2. The Iterative Solver

First of all, let us remark that boundary condition (17), which couples permeation and polarization of concentration is nonlinear. Any rigorously implicit computation therefore requires an iterative solver for solving the Prandtl system of Section 3. More precisely, at the channel section $z^{n+1},$ we are faced with two coupled systems very similar to the heat equation, and an iterative method is required to perform their decoupling. Furthermore, this is our numerical experience that the coupling between permeation velocity $U\equiv u_{J}^{n+1}$ and polarization is a keystone for the accuracy of any numerical method applied to fouling.

Since permeation velocity is involved in a nonlinear boundary condition, its assessment will be obtained as the limit of an iterative process where $U^{k}$, k=0,1,2…, represents a series of estimates. At convergence of the iterative process, we shall set $u_{J}^{n+1}=lim\left(U^{k}\right)$. More precisely, in ODEs (21) and (22), the iterative solver considers the quantities without ($\hat{\;\;}$) as unknowns related to the new iteration (say *k*), while the quantities labelled with ($\hat{\;\;}$) are those already known. In the description of the iterative solver that follows, the unknowns $\left\{C_{j}^{n+1},\;w_{j}^{n+1},\;u_{j}^{n+1},\;j=0,\ldots ,J\right\}$ are temporarily denoted $\left\{C_{j}^{k},\;w_{j}^{k},\;u_{j}^{k},\;j=0,\ldots ,J\right\}$, since they are computed at each iteration, and will be labelled with superscript “n+1” only at convergence.

The final numerical system to be solved incorporates a discretization of the transverse differential or integration operators. First and second transverse derivatives are approximated by the centred finite difference operators of the second order. As for carrying out the extrapolation step, we select the Adams–Bashforth second order scheme (i.e., ${\hat{w}}_{j}^{n+1}=2w_{j}^{n}-w_{j}^{n-1}$), while the Simpson rule is used for determining the $\chi_{j}$. The initial guess of the iterative solver is provided by an extrapolation step, which gives us the initial set $\left\{C_{j}^{k=0},\;w_{j}^{k=0},\;u_{j}^{k=0},\;j=0,\ldots ,J\right\}$.

Although the overall numerical scheme is fully implicit, the left-hand sides of Equations (21) and (22) become linear and are treated implicitly (i.e., unknown at iterative step *k*, while the right-hand side is computed explicitly (i.e., with values known at step k−1). More precisely, in Equations (21) and (22), all the quantities labelled with the superscript “$\hat{\;}$” are those of step k−1. We then obtain two decoupled linear systems: for the concentration field, the system is tri-diagonal, while, for the axial velocity field, the linear system is an essentially tri-diagonal system as described below to be solved for computing a new estimate of the transverse profiles of the axial velocity in the section $z^{n+1}\equiv \left(n+1\right)\Delta z$. At boundary node j=0, the symmetry condition closes the first row of the tri-diagonal system by setting $C_{j=-1}^{k}=C_{j=1}^{k}$. As for the closure of the last row (j=J), it depends on the situation with respect to fouling, as follows.

We now describe the general process characterizing the iteration at step *k*. The first stage of the iteration *k* considers the computation of the concentration, which is carried out with respect to ODE (21) equipped with the Robin boundary condition $Pe_{in}U^{k-1}C_{J}^{k}{-\partial C/\partial x|}_{J}^{k}=0$ at node $x_{J}=1$. This operation provides us with the solute concentration field. Then, two situations can arise:

(a) If $C_{J}^{k}\leq N_{d}$, no fouling is involved at section $z^{n+1}$. Then, the computation of the axial velocity profile $\left\{w_{j},j=0,\ldots ,J-1\right\}$ is undertaken with a tri-diagonal system complemented with an additional (full) row composed of constraint (24), since the pressure unknown $p^{k}$ (with $p^{n+1}=lim\left(p^{k}\right)$) increases the number of velocity unknowns by one more unit. Simultaneously, Equation (22), which implies $p^{k}$, gives rise to an additional last column, which is also full. The algorithm for solving this non-trivial—but essentially tridiagonal—-linear system will be described elsewhere. At the end of this stage, we possess a new estimate of the concentration and axial velocity profiles complemented with the value $p^{k}$ on pressure.

(b) If $C_{J}^{n+1}>N_{d}$, the polarization of concentration becomes over-saturated (or supercritical), and some fouling must occur. At steady state (i.e., when the deposit has stopped), the critical concentration $N_{d}$ must be recovered at the membrane (knowing that the material deposit adapts in such a manner that the resulting permeation satisfies the mass transfer at the wall). Hence, the concentration profile is computed again by solving Equation (21) with Dirichlet conditions $C_{J}^{k}=N_{d}$ at node $x_{J}=1$. The concentration profile being known, the Robin condition $Pe_{in}U^{k}C_{J}^{k}{-\partial C/\partial x|}_{J}^{k}=0$ at node $x_{J}=1$ allows us to compute the permeation $U^{k}$ that satisfies the mass transfer at the membrane. Now, the constraint (23) offers an alternative closure to ODE (22), which is solved with the same algorithm as in case (a). Similarly to case (a), this stage ends up with iteration *k*, since new concentration and axial velocity profiles, complemented with the value $p^{k}$ of the pressure, have been determined.

When following either path (a) or path (b), the process reaches the stage where an estimate of the axial velocity profile is available. From this profile, the transverse integration of the incompressibility constraint allows us to compute the transverse velocity profile $u_{j}^{k}$. We are hence ready to start a new iterative step by setting “(k−1)⟵k”. This process is renewed until a satisfactory convergence $U^{k=K}$ is attained concerning the series $\left\{U^{0},\;U^{1},\ldots .,\;U^{k=K}\equiv u_{J}^{n+1}\right\}$.

**Remark** **4.** *As soon as some deposit has occurred in the channel section $z^{n+1}=\left(n+1\right)\Delta z$, the permeation velocity only depends on the overall mass transfer, and in particular on the mass budget at the membrane. The role of the Starling–Darcy constraint (24) is reduced to adapt the new membrane resistance (and the corresponding deposit) to the local pressure and osmotic effects. More precisely, the new local membrane resistance at position $z^{n+1}$ is assessed at a convergence of the iterative process, and reads*

(26) $$\frac{I(z^{n+1})}{I_{0}}=\frac{p^{n+1}-N_{osm}C_{J}^{n+1}}{u_{J}^{n+1}}.$$

## 5. Numerical Investigation on Reversible Fouling

Since the overall problem depends on seven independent numbers, the present numerical experiments consist of fixing certain numbers and varying the others. As previously discussed, the membrane number $\beta \equiv \mu_{0}/\left(I_{0}d\right)$ has been set to zero very early in our analysis. Because we want to focus on fouling, we have decided to neglect pressure drop and osmotic (counter)-effects. This is why number $\alpha \equiv {\left(\mu_{0}I_{0}/d\right)}^{1/2}W_{in}/P_{in}$ is now set to a small value, say $10^{-2}$. As for the osmotic number $N_{osm}\equiv iRTC_{in}/P_{in}$, we decide to also select a small value (say $5\times 10^{-3}$) that will only serve as a marker of high polarization. In other words, in the absence of extreme polarization, both assumptions on α and $N_{osm}$ allow us to consider that the net operating pressure is nearly constant.

Now, in the absence of fouling, both assumptions have a simple consequence: permeation is more or less constant. Thus, as in a nearly “clean water” experiment, axial flow exhaustion will be found at about $L=L_{de}$ (or at about λ=1 in non-dimensional form). Therefore, in the possible presence of fouling (which will delay axial flow exhaustion), it is meaningful to at least study the problem in the parameter range 0≤λ≤1 (and obviously for 0≤z≤λ≤1 ). At the channel entrance, the feed flow profile is supposed to be a Hagen–Poiseuille type and the concentration profile is uniformly set to $C_{in}$, or 1 in non-dimensional form.

### 5.1. Description of the Fouling Onset

**Figure 2:**
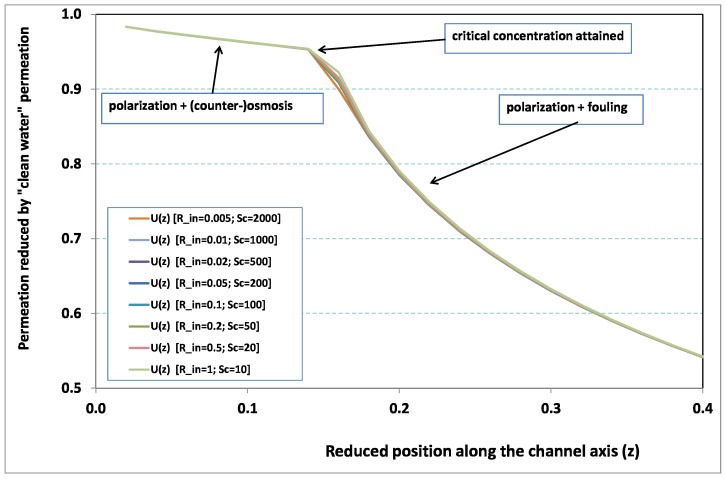
Permeation vs. axial position for various Reynolds numbers for Péclet number fixed to $Pe_{in}=10$ ($N_{d}=10$).

**Figure 3:**
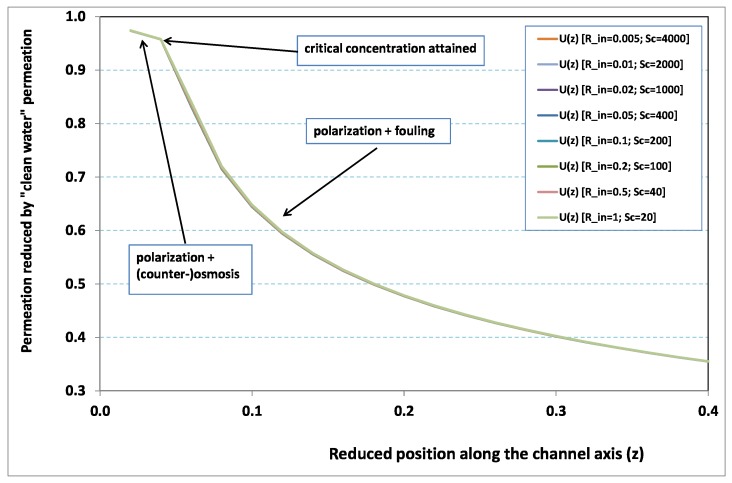
Permeation vs. axial position for various Reynolds numbers for Péclet number fixed to $Pe_{in}=20$ ($N_{d}=10$).

Within the framework of the Berman theory, which treats of channel flow with uniform leakage, any pressure change along the axis is found to be on the order of the quantity $-\alpha^{2}K\left(R_{in}\right)$, where $K(R_{in})$ is the so-called Berman constant. Since $K(R_{in})$ has a limited range of variation for the standard filtration configurations (say $0\leq K(R_{in})\leq 3$), any variation of $R_{in}$ will have a negligible effect on pressure as long as α≪1. In other words, at steady state, hydraulic effects are expected to be small in comparison with mass transfer effects. This analysis is corroborated by Figure 2 and Figure 3, where local permeation is drawn against the axial position in the channel. Note that permeation is normalized by the “clear water” permeation $U_{in}$, whereas axial position is reduced by the “clear water” exhaustion length $L_{de}$. In both figures, any variation of $R_{in}$ does not modify the curves, provided that both parameters $R_{in}$ and Sc are varied in such a manner that their product, the transverse Péclet number $Pe_{in}\equiv R_{in}Sc$, remains identical for all curves. Figure 2 [resp. Figure 3] concerns $Pe_{in}=10$ [resp. $Pe_{in}=20$]. In each figure, all curves superimpose nearly perfectly. In other words, the relevant parameter is $Pe_{in}$, and not the pair $\left\{R_{in},Sc\right\}$. This remark induces a significant gain for the present analysis, since the remaining parameters to be investigated are now reduced to $\left\{Pe_{in},\;N_{d},\;\lambda \right\}$.

In Figure 2 and Figure 3, permeation starts with a value close to 1 (i.e., nearly the permeation of “clean water”, because polarization of concentration and the subsequent osmotic (counter-)effects are low). Then, the solute boundary layer develops along the membrane inner surface and polarization arises. The increasing polarization along the downstream direction is indicated by the osmotic effects that lower the permeation (slightly, since the osmotic number has been chosen feeble). Farther along the axis, polarization of concentration reaches the critical concentration at the membrane, fouling occurs, and permeation hindrance becomes significant (provoking a rapid change in the permeation curve slope). We additionally observe that increasing $Pe_{in}$ enhances polarization and the onset of fouling occurs earlier in the channel. To summarize, the present numerical experiments corroborate the fact that $R_{in}$ does not play the leading part in such a permeation system (steady laminar flow and α≪1, provided). By contrast, $Pe_{in}$, the transverse Péclet number, controls the locus where fouling starts to take place, as well as the permeation under fouling.

### 5.2. Mass Transfer Controls Permeation

**Figure 4:**
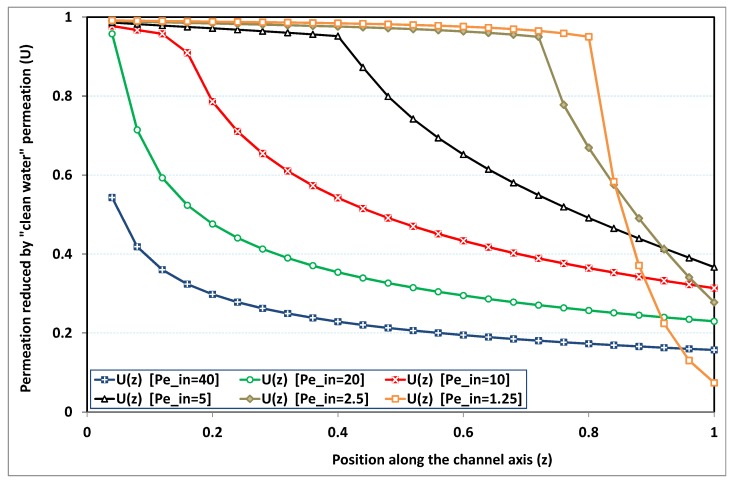
Permeation vs. axial position for $N_{d}=10$ and various $Pe_{in}$.

**Figure 5:**
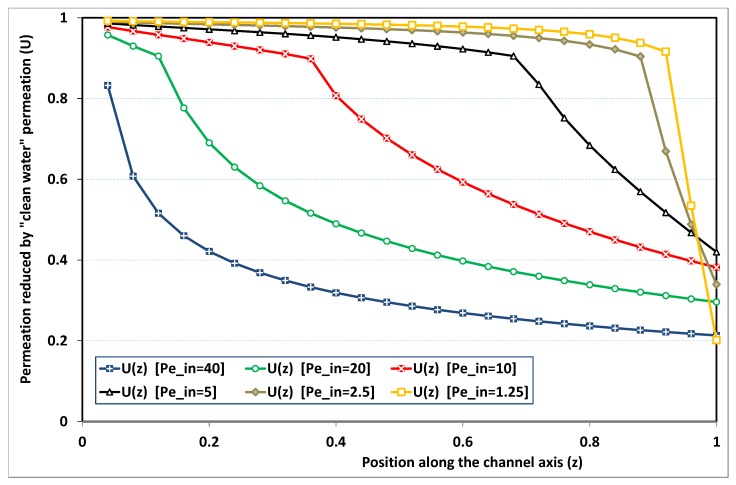
Permeation vs. axial position for $N_{d}=20$ and various $Pe_{in}$.

As a consequence of the previous paragraph, only the variation of $Pe_{in}\equiv Pe_{in}d{\left(I_{0}D_{0}\right)}^{-1}$ will be considered hereinafter, whereas $R_{in}$ and Sc will no longer be seen as independent parameters. Figure 4 and Figure 5 investigate the role played by $Pe_{in}$ on permeation for two different critical concentrations characterized by the deposit numbers $N_{d}=10$ and $N_{d}=20$, respectively.

In Figure 4, permeation is plotted for $N_{d}=10$, which is a standard value for the deposit number: it particularly corresponds to sea water desalination, where salt concentration is around 35 kg/m${}^{3}$, while salt maximum solubility in water is about 350 kg/m${}^{3}$ at standard conditions. At high transverse Péclet number, a high level of polarization is rapidly obtained at the channel entrance, so that the permeation strength of clean water never occurs. On the other hand, if the transverse Péclet number is low, permeation remains close to that of clean water in the largest part of the channel. However, as the bulk solute concentration increases (due to solvent leakage) when approaching the dead-end length, the deposit (or saturation) concentration is reached and the phenomenon of fouling makes the permeation rapidly diminishing (in the absence of fouling and osmotic effects, we recall that permeation would stop at z=1). For intermediate Péclet numbers, fouling arises earlier in the channel. There is a transitional value of $Pe_{in}$, for which fouling occurs immediately at the start of the channel (say here for $Pe_{in}>20$).

Note that the case of high polarization obtained for $P_{in}=40$ clearly stands beyond the validity domain of the Prandtl approximation, since a strong discontinuity occurs at the channel entrance. Evidently, axial diffusion cannot be neglected at this point. A crude use of the present analysis for $P_{in}\geq 40$ would hence underestimate permeation.

When $N_{d}$ is chosen higher, the previous general trend is maintained. Comparing Figure 4 and Figure 5, we note that the upper four curves remains strictly identical as long as no fouling appears. The difference lies in the fact that the fouling events of Figure 4 occurred sooner in the channel. As an illustration, the highest curve in Figure 5 (obtained for the lowest $Pe_{in}$) indicates that fouling here arises farther, close to the dead end length (of clean water permeation). In the same manner, all the curves of Figure 5 show a better permeation due to a delayed occurrence of fouling. By contrast with the previous value of $N_{d}$ (i.e., $N_{d}=10$), the case with $N_{d}=20$ exhibits results that are situated in the validity domain of the Prandtl approximation, except the case $Pe_{in}=40$ which nevertheless presents a rather limited discontinuity at entrance.

To sum up, two intuitive features have been confirmed in this paragraph:

- increasing $Pe_{in}$, the transverse Péclet number, enhances polarization of concentration, making the occurrence of fouling earlier and reducing the overall permeation.
- increasing $N_{d}$, the deposit (or saturation) number, delays the occurrence of fouling, and enhances the overall permeation.

To account for both opposite trends, the idea followed in the next paragraph consists of introducing a new number (hereafter called fouling number), built as the ratio of the transverse Péclet number to the deposit number. More precisely, the new number reads:

(27) $$F_{l}\equiv \frac{Pe_{in}}{N_{d}}=\frac{P_{in}C_{in}d}{I_{0}D_{0}C_{d}}.$$

What follows consists of performing a new sorting of the various permeation computations (as those described previously) for various pairs $\left\{Pe_{in},\;N_{d}\right\}$. The new classification will next affirm the major role played by $F_{l}$.

### 5.3. Role of Fouling Number $F_{l}$

**Figure 6:**
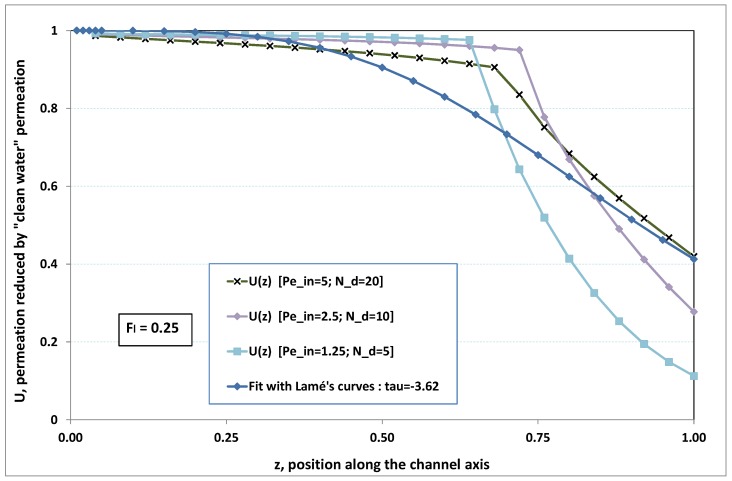
Permeation vs. axial position for various pairs $\left\{Pe_{in},N_{d}\right\}$ such that $F_{l}=Pe_{in}/N_{d}=0.25$.

**Figure 7:**
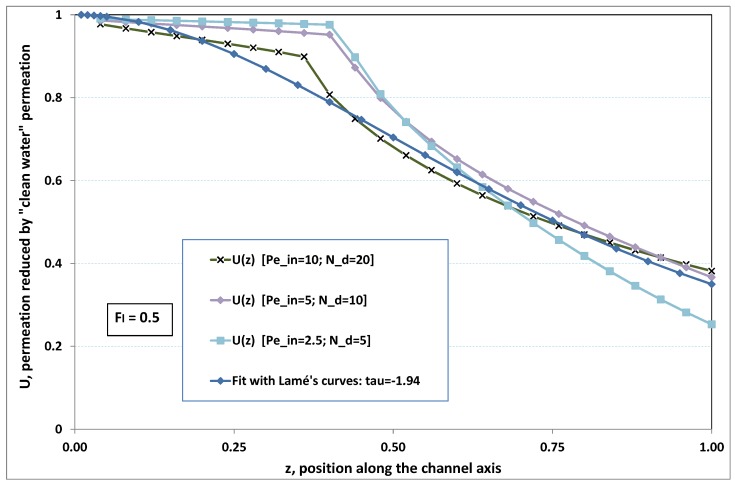
Permeation vs. axial position for various pairs $\left\{Pe_{in},N_{d}\right\}$ such that $F_{l}=Pe_{in}/N_{d}=0.5$.

**Figure 8:**
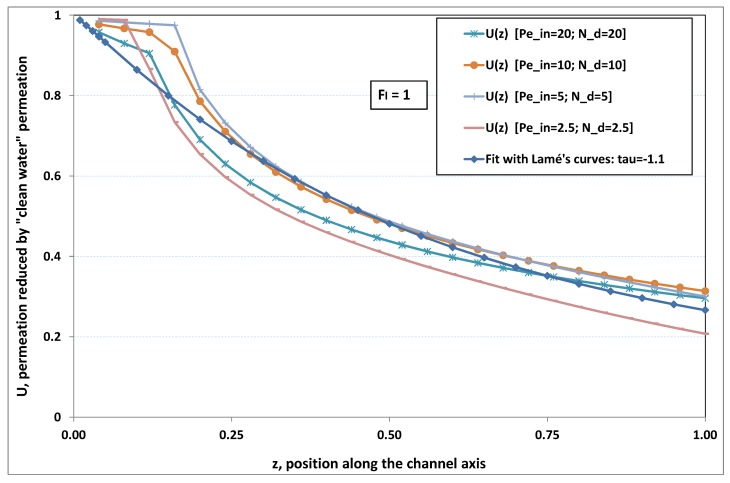
Permeation vs. axial position for various pairs $\left\{Pe_{in},N_{d}\right\}$ such that $F_{l}=Pe_{in}/N_{d}=1$.

**Figure 9:**
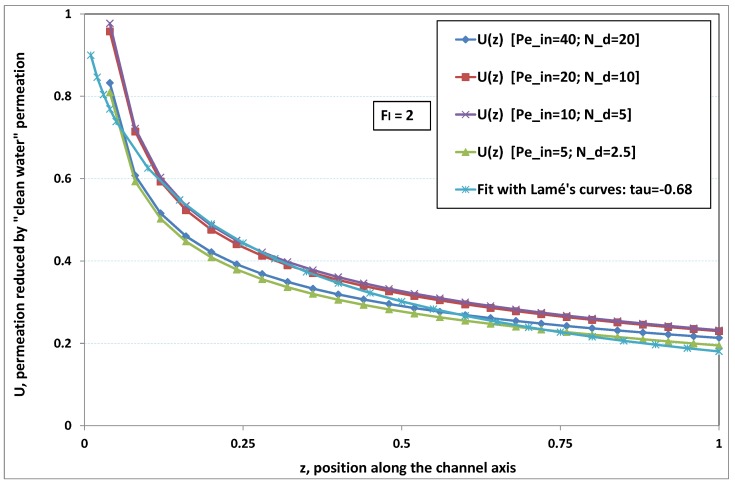
Permeation vs. axial position for various pairs $\left\{Pe_{in},N_{d}\right\}$ such that $F_{l}=Pe_{in}/N_{d}=2$.

**Figure 10:**
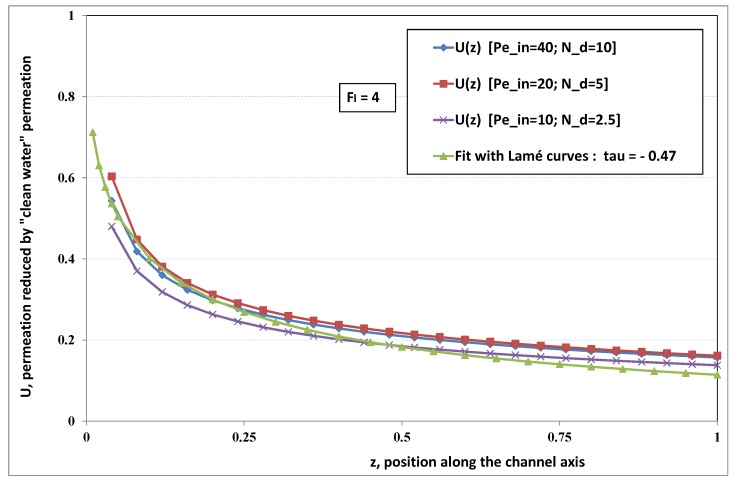
Permeation vs. axial position for various pairs $\left\{Pe_{in},N_{d}\right\}$ such that $F_{l}=Pe_{in}/N_{d}=4$.

The point developed in the present paragraph concerns the claim that the single number $F_{l}$ plays the leading role on permeation. To demonstrate this point, we consider all our numerical results on local permeation, and in the same figure we gather the curves that correspond to a given $F_{l}$. We obtain the Figure 6, Figure 7, Figure 8, Figure 9 and Figure 10. The classification of these figures follows an increasing sorting of $F_{l}$, namely $F_{l}=\;0.25,\;0.5,\;1,\;2,\;4$. In every figure, the curves are plotted for various pairs $\left\{Pe_{in},\;N_{d}\right\}$, the ratio of which gives the same value of $F_{l}$. We observe that, in a given figure, all curves follow an identical general trend. Note that an additional curve is plotted in each figure: it corresponds to an attempt for fitting the numerical results by a particular family of analytical curves; this is the aim of Section 6.

On the one hand, when $F_{l}$ increases from 0.25 to 1, fouling conditions are attained earlier and earlier in the channel. In the first channel part, polarization grows downstream of the channel, and produces a slight drop in permeation owing to the non-zero osmotic number. This decline nevertheless remains insignificant, since the osmotic number has been chosen to be very low (as well as pressure drop). When the deposit conditions are attained at the channel wall, fouling occurs. The occurrence of fouling implies a rapid change in the slope of the permeation curves. This change is all the more rapid that fouling arises lately in the channel (because the bulk concentration is already high). More precisely, for $F_{l}\ll 1$, fouling hardly occurs and the permeation curves remain slowly decreasing straight lines up to the vicinity of the dead-end length. At such a point (which is situated close to the locus of exhaustion for the “clear water” flow), the concentration in the retentate is, however, very high, since most of the solvent has permeated. It is clear that our model with constant solute diffusion locally here again leaves its validity domain, i.e., when the solution’s concentration is too high.

On the other hand, for large values of $F_{l}$ (say, $F_{l}=\;1,\;2,\;4$, see Figure 8, Figure 9 and Figure 10), we are faced with a series of curves, again very close to each other in every figure. The curvature of these curves is more or less everywhere positive. Fouling appears in a region close to the channel entrance, since polarization of concentration becomes rapidly high, or the critical concentration is easy to reach. Hence, the concentration for deposit is very early attained in the channel. From a quantitative point of view, the results of Figure 10 must certainly be considered as inaccurate, since the Prandtl model fails to treat the channel very entrance, where a discontinuity in concentration is computed. Hence, the results for $F_{l}\geq 4$ cannot be considered as consistent with the Prandtl assumptions.

## 6. Complementary Analytical Treatment of Reversible Fouling

At this point of our analysis, we have drastically reduced the number of the relevant parameters: in a certain validity range of our model to be specified later, two parameters $F_{l}$ and λ now allow us to analyse the properties of the present model of reversible fouling. We shall take profit from such an opportunity to try to gather all the numerical results within a simple analytical expression. This stage corresponds to the following procedure of fit.

### 6.1. Analytical Fit of Critical Length

In all figures from Figure 4, Figure 5, Figure 6, Figure 7, Figure 8 and Figure 9, the permeation curves exhibit a point of abscissa where permeation starts to sharply decline. The literature calls this point “critical abscissa” or “critical length” of the membrane device, here denoted $z_{c}$. Note that, in Figure 10, there is no value for $z_{c}$, since the computation indicates that fouling occurs at the channel entrance straightaway. Hence, the results of Figure 10 present a discontinuity at z=0 and the corresponding parameters do not satisfy the assumptions of the Prandtl approximation.

**Figure 11:**
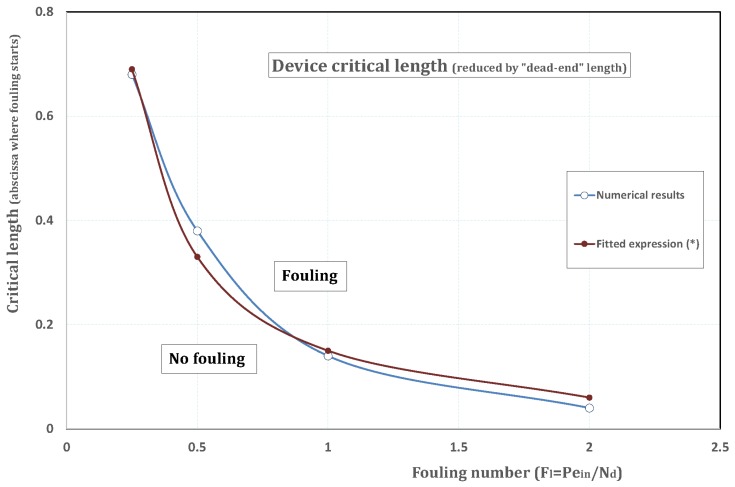
$z_{c}$, the channel critical length vs. $F_{l}\equiv Pe_{in}/N_{d}$. Below both curves, the channel is too short and fouling never occurs; (*) fitted curve corresponds to expression (28).

Four averaged values of $z_{c}$ have been assessed from Figure 6, Figure 7, Figure 8 and Figure 9 and have been reported in Figure 11 as a function of $F_{l}$. For the range $\left\{0.25\leq F_{l}\leq 2\right\}$, the following analytical fit has been estimated for $z_{c}$

(28) $$z_{c}=0.06+0.18\left[F_{l}^{-1}-0.5\right]$$

or in dimensional form

(29) $${\tilde{z}}_{c}=\frac{W_{in}I_{0}d}{P_{in}}\left\{0.06+0.18\left[\frac{C_{d}I_{0}D_{0}}{C_{in}P_{in}d}-0.5\right]\right\}.$$

If *L*, the actual device length, is less than ${\tilde{z}}_{c}$, no fouling occurs within the membrane.

### 6.2. Critical Flux of Permeation

Hence, setting ${\tilde{z}}_{c}=L$, expression (29) gives access to an expression of $\varphi_{c}$, the so-called critical flux, which corresponds to the flux (i.e., $\varphi =P_{in}/I_{0}$) at which fouling starts to occur. Then, $\varphi_{c}$ is the solution of

(30) $$\frac{L}{d}=\frac{W_{in}}{{\tilde{\varphi }}_{c}}\left\{0.06+0.18\left[\frac{C_{d}D_{0}}{{\tilde{\varphi }}_{c}C_{in}d}-0.5\right]\right\},$$

which obviously reads

(31) $${\tilde{\varphi }}_{c}=0.36N_{d}\frac{D_{0}}{d}{\left\{0.03+\sqrt{9.10^{-4}+0.72N_{d}\frac{D_{0}L}{W_{in}d^{2}}}\right\}}^{-1}.$$

### 6.3. Analytical Fit of Local Permeation

As can be seen in Figure 6, Figure 7, Figure 8, Figure 9 and Figure 10, an additional curve is plotted in every figure. It corresponds to an analytical fit with a family of curves, which experience a change in concavity accordingly with the value of a single parameter, and which possesses a straightforward primitive integral. A good candidate is the first derivative of the family of Lamé’s curves with τ as a single parameter. Lamé’s family is defined as $L_{\tau }\left(z\right)={\left(1+z^{\tau }\right)}^{1/\tau }$. More precisely, we search after an expression of the permeation in the following form:

(32) $$U\left(x=1,z\right)\approx L_{\tau }^{\prime }\left(z\right)\equiv {\left(1+z^{-\tau }\right)}^{(1-\tau )/\tau }.$$

At the origin z=0, $L_{\tau }^{\prime }\left(z\right)$ exhibits a change in second derivative, which occurs for about τ=−1.1. This property was sought, since, accordingly with Figure 6, Figure 7, Figure 8, Figure 9 and Figure 10, we had to find a family of curves with a single parameter that exhibits a change in concavity as $F_{l}$ changes. The point is now to relate the fitted values of τ that appear in Figure 6, Figure 7, Figure 8, Figure 9 and Figure 10 with the corresponding value of $F_{l}$, the fouling number. Elementary linear regression between τ et $F_{l}^{-1}$ gives the following relationship between τ et $F_{l}$

(33) $$\tau =-(0.26+\frac{0.84}{F_{l}}).$$

In regard to Figure 6, Figure 7, Figure 8, Figure 9 and Figure 10, we admit that the overall fit may have a limited quality, particularly when considering that the various numerical data gathered in the same figure have some scattering. It is nevertheless interesting that a single-parameter family can catch the general trends. Furthermore, the discrepancies observed in these figures will be improved by integration when we shall consider the recovery ratio as a function of λ. To sum up, even though the fitting stage is not immune from criticism, possessing a mathematical expression will allow us to easily handle a large amount of numerical results.

### 6.4. Fouling Resistance to Permeation

The additional resistance to permeation resulting from fouling can be deduced from the analysis of the local permeation. We have underlined the fact that—in steady state—material deposit stops when the mass transfer balance is attained. In other words, hindrance to permeation stops developing when the transverse mass transfer at the membrane is reduced enough to be equilibrated by the (back-)diffusion towards the bulk. Let us define $I_{f}\left(z\right)$, the additional resistance to permeation due to material deposit at the axial position *z*. If we neglect the (counter-)osmotic effects and the viscous pressure drop, the permeation velocity can easily be written as

(34) $$\tilde{U}\left(x=1,z\right)=\frac{P_{in}}{I_{0}+I_{f}\left(z\right)}=\frac{U_{in}}{1+I_{f}\left(z\right)/I_{0}}.$$

Hence, the data from Figure 6, Figure 7, Figure 8, Figure 9 and Figure 10 allow us to assess the resistance to filtration that fouling creates at the membrane inner surface. More precisely, the previous fit provides us with the following analytical expression

(35) $$I_{f}\left(z\right)=I_{0}\left({\left[1+z^{-\tau }\right]}^{\frac{\tau -1}{\tau }}-1\right),$$

where $\tau =-0.26-0.84/F_{l}$. The latter expression reminds us that the additional resistance due to fouling is not uniform along the membrane.

### 6.5. Analytical Expression of Recovery Ratio

The paragraph is devoted to assess the total amount of purified fluid (or permeate). This consists of integrating our local data to get a numerical prediction of $R_{ec}$, the recovery ratio (or factor) as a function of λ, the channel length reduced by the dead-end length. In the case of a “clear water” flow, let us recall that $R_{ec}\left(\lambda \right)=\lambda$ with 0≤λ≤1, since the local permeation would be equal to 1 everywhere, and the axial flow exhausted for λ=1.

When some hindrance to permeation arises, it is meaningful to investigate certain values of λ larger than 1. In the experimental practice, however, λ is generally chosen to be smaller than 1. Hence, the paragraph considers the numerical results for 0≤z≤λ≤1 only. To obtain the recovery factor, the axial integration of the local permeation is required. The integration of expression (32) obviously gives the Lamé’s function

(36) $$R_{ec}\left(\lambda \right)={\left(1+\lambda^{\tau }\right)}^{\frac{1}{\tau }}\;\;\mathrm{with}\;\tau =-0.26-\frac{0.84}{F_{l}}.$$

**Figure 12:**
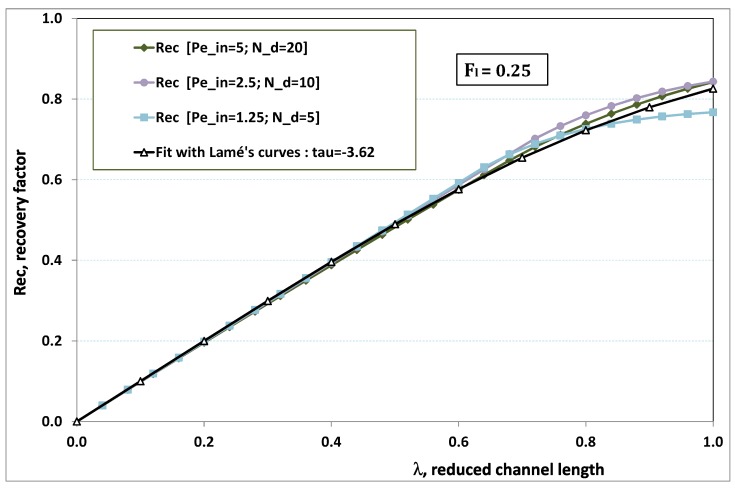
Recovery factor vs. channel reduced length for various pairs $\left\{Pe_{in},N_{d}\right\}$ such that $F_{l}\equiv Pe_{in}/N_{d}=0.25$.

**Figure 13:**
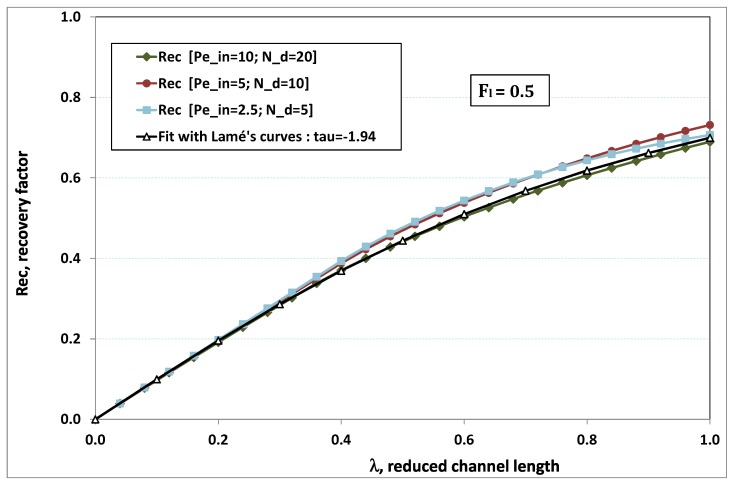
Recovery factor vs. channel reduced length for various pairs $\left\{Pe_{in},N_{d}\right\}$ such that $F_{l}\equiv Pe_{in}/N_{d}=0.5$.

**Figure 14:**
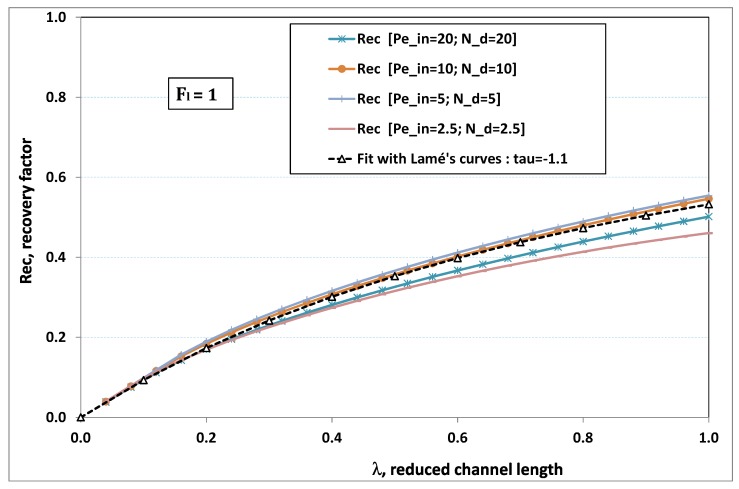
Recovery factor vs. channel reduced length for various pairs $\left\{Pe_{in},N_{d}\right\}$ such that $F_{l}\equiv Pe_{in}/N_{d}=1$.

**Figure 15:**
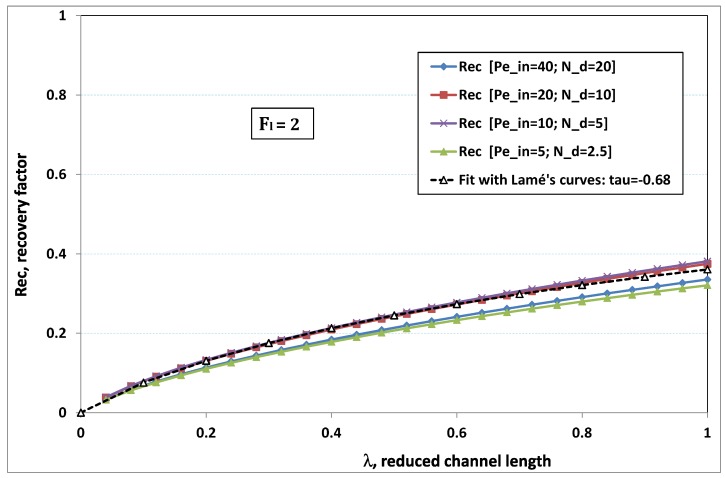
Recovery factor vs. channel reduced length for various pairs $\left\{Pe_{in},N_{d}\right\}$ such that $F_{l}\equiv Pe_{in}/N_{d}=2$.

**Figure 16:**
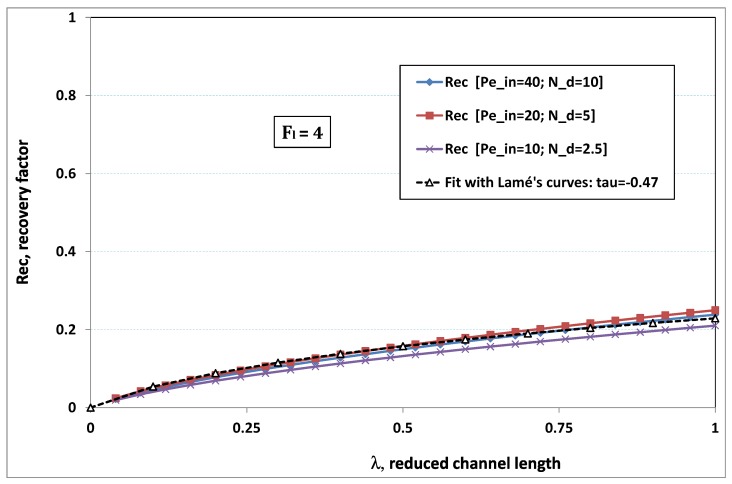
Recovery factor vs. channel reduced length for various pairs $\left\{Pe_{in},N_{d}\right\}$ such that $F_{l}\equiv Pe_{in}/N_{d}=4$.

To assess the quality of analytical expression (36) regarding the recovery factor obtained numerically, we have drawn the numerical results with respect to λ, the reduced channel length, for various $F_{l}$. The five cases previously considered have been investigated again, and the comparison with expression (36) is conducted in Figure 12, Figure 13, Figure 14, Figure 15 and Figure 16, where recovery ratio is plotted against channel length for various $F_{l}$.

In Figure 12, Figure 13, Figure 14, Figure 15 and Figure 16, we observe that the estimate proposed by the fit gives a much better assessment for the numerical recovery factor. The reason is due to the integration process, which smoothes the various discrepancies. Provided that $N_{d}>2$ and $Pe_{in}\leq 40$ hold, it is hence tempting to claim that the difference between fit and numerical predictions lie in standard error bars affecting any experimental measurement.

### 6.6. Fouling Rate

Let us now seek for a manner of qualifying the hindrance to permeation that results from fouling. The reduction in permeation obviously depends on the channel length, since fouling can arise at any distance from the channel entrance. Consequently, let us consider the relative difference between the ideal recovery (i.e., obtained in the situation of clear water filtration) and the actual recovery when fouling arises. This quantity denoted $R_{f}$ and called fouling rate, is defined as follows:

(37) $$R_{f}\left(\lambda ,F_{l}\right)=\frac{\lambda -{\left(1+\lambda^{\tau }\right)}^{1/\tau }}{\lambda }=1-{\left(1+\lambda^{-\tau }\right)}^{1/\tau },\;\;\mathrm{with}\;\tau =-(0.26+\frac{0.84}{F_{l}}).$$

**Figure 17:**
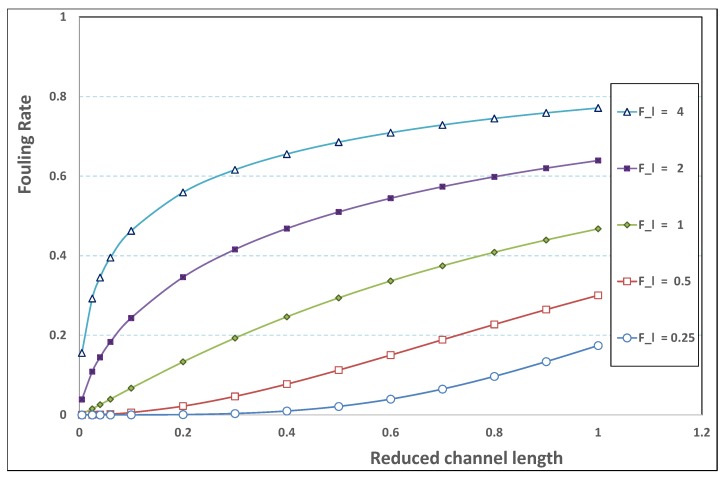
Fouling rate vs. channel reduced length for various values of $F_{l}$.

Figure 17 presents the variations of expression (37) as a function of the reduced channel length λ for various fouling numbers $F_{l}$. In Figure 17, the general trend is retrieved: as the fouling number increases, the strength of the fouling hindrance is enhanced. Moreover, one recovers the change in concavity for $F_{l}\approx 1$. This recalls that, for $F_{l}<O\left(1\right),$ fouling occurs lately in the channel, while, for $F_{l}>O\left(1\right),$ fouling arises close to the channel entrance. In the latter case, the membrane filtration rapidly loses its efficiency, due to the early fouling occurrence in the channel.

### 6.7. Sustainable Flux

This invites us to consider some critical value $F_{l}^{s}=O\left(1\right)$ separating both regimes: (a) at low $F_{l}$, fouling might occur, but does not greatly affect the membrane efficiency; (b) at high $F_{l}$, fouling plays a major role, and drastically reduces the permeation, rendering the device much less efficient. In that sense, $F_{l}^{s}$ should be compared to the concept of “sustainable flux” [6], as follows: for a given channel length, up to a certain critical value $F_{l}^{s}$, the fouling rate is considered as acceptable accordingly with Figure 17. Then, the existence of $F_{l}^{s}$ corresponds to a certain permeation that reads

(38) $$\left\{F_{l}=F_{l}^{s}\right\}\;\Leftrightarrow \;\left\{Pe_{in}^{s}=F_{l}^{s}N_{d}\right\}\;\Leftrightarrow \;\left\{P_{in}^{s}=\frac{F_{l}^{s}N_{d}D_{0}I_{0}}{d}\right\}.$$

Therefore, for fixed experimental conditions, the sustainable flux will correspond to the flux obtained with the inlet pressure given by expression (38). This flux can be derived from expression (36), or from the dimensional form (42) of the next section.

## 7. Dimensional Interpretation

For a given experimental situation, the standard parameters that are generally varied are $P_{in}$, the transmembrane pressure, $C_{in}$, the feed concentration, and $W_{in}$, the mean inlet velocity of the feed flow. Since both $P_{in}$ and $W_{in}$ enter in the definition of λ, we need to return to a dimensional interpretation for separating the role of the different parameters. Before leaving the dimensionless forms, let us stress on the merits of Dimensional Analysis, which here allowed us to drastically reduce the number of the relevant parameters and conduct a nearly exhaustive analysis.

### 7.1. Dimensional Expression of Permeation Flux

Let us now express $R_{ec}$, the recovery ratio given in expression (36), in terms of physical quantities. This gives

(39) $$R_{ec}={\left[1+{\left(\frac{P_{in}L}{W_{in}I_{0}d}\right)}^{\tau }\right]}^{\frac{1}{\tau }}\;\mathrm{with}\;\tau =-0.26-0.84\frac{C_{d}I_{0}D_{0}}{C_{in}P_{in}d}.$$

In the “clear water” limit case, τ tends to −∞. When λ<1, we easily retrieve that the limit of expression (39) is

(40) $$R_{ec}\to \lambda =\frac{P_{in}L}{W_{in}I_{0}d}\;\;\mathrm{as}\;\tau \to -\infty .$$

Let us now denote by $\varphi_{p}$, the (overall) flux of permeation which is obtained for a given filtration system. In the case of a “clear water” flow, the permeation flux per surface unit of membrane (denoted $\varphi_{p}\left(C_{in}=0\right)$) is simply provided by

(41) $$\varphi_{p}\left(C_{in}=0\right)=\frac{P_{in}}{I_{0}}.$$

With the use of $R_{f}$, the fouling rate given by expression (37), we obtain the permeation flux per unit of membrane surface as

(42) $$\begin{array}{ccc} \varphi_{p} & = & \left\{1-R_{f}\left(\lambda ,F_{l}\right)\right\}\frac{P_{in}}{I_{0}}={\left\{1+{\left(\frac{P_{in}L}{W_{in}I_{0}d}\right)}^{-\tau }\right\}}^{1/\tau }\frac{P_{in}}{I_{0}}\;,\\ \mathrm{with}\;\tau & = & -0.26-0.84\frac{C_{d}I_{0}D_{0}}{C_{in}P_{in}d}. \end{array}$$

Formula (42) gathers all previous numerical results ($Pe_{in}\leq 40$ and $N_{d}>2$ provided) and gives them in dimensional form. This expression can be directly compared with the experiments. From expression (42), interesting general trends observed in the experiments can be retrieved, as described in the following paragraph.

Regarding the permeate flux, there are several experimental measurements often provided in the literature: (a) the filtration flux against $P_{in}$ to exhibit the role of pressure on polarization and subsequent fouling: (a1) for various fixed values of $W_{in}$, or (a2) for different $C_{in}$. Measurement (a1) are devoted to illustrate the part played by the “shear at the membrane surface”, while cases (a2) search for the role of the feed initial concentration on permeation. Another frequent measurement is (b) the filtration flux against $C_{in}$ to point out the role of the feed concentration level on fouling. Therefore, the purpose of the next paragraph consists of varying the following three parameters $\left\{P_{in},W_{in},C_{in}\right\}$, and drawing the curves related to cases (a1), (a2) and (b).

### 7.2. Discussing the Roles of the Main Parameters

First of all, varying $\left\{P_{in},W_{in},C_{in}\right\}$ consists of fixing the other parameters. To keep the highest level of generality, let us imagine an experiment of reference, carried out in a range of parameters that does not lead to fouling. This experiment of “clear water” type has both pressure ${\left(P_{in}\right)}_{0}$ and concentration ${\left(C_{in}\right)}_{0}$ low enough that the fouling number is low, say ${\left(F_{l}\right)}_{0}=0.25$. On the other hand, a moderate value has to be chosen for ${\left(W_{in}\right)}_{0}$ because the exhaustion length must remain much larger than the actual channel length. Let us choose ${\left(W_{in}\right)}_{0}$ such that ${\left(\lambda \right)}_{0}=0.5$. We additionally denote the permeation flux that results from this experiment by ${\left(\varphi_{p}\right)}_{0}$, which is nothing but ${\left(P_{in}\right)}_{0}/I_{0}$.

From this experiment of reference, we now use the set of units, namely $\left\{{\left(P_{in}\right)}_{0},{\left(W_{in}\right)}_{0},{\left(C_{in}\right)}_{0}/C_{d},{\left(\varphi_{p}\right)}_{0},{\left(F_{l}\right)}_{0}=0.25,{\left(\lambda \right)}_{0}=0.5\right\}$, to reduce the three main parameters and the permeation flux. Therefore, from Equation (42), we write the normalized permeation flux, as follows:

(43) $$\begin{array}{ccc} \frac{\varphi_{p}}{{\left(\varphi_{p}\right)}_{0}} & = & {\left\{1+{\left({\left(\lambda \right)}_{0}\frac{{\left(W_{in}\right)}_{0}}{W_{in}}\frac{P_{in}}{{\left(P_{in}\right)}_{0}}\right)}^{-\tau }\right\}}^{1/\tau }\frac{P_{in}}{{\left(P_{in}\right)}_{0}},\;\\ \mathrm{with}\;\tau & = & -0.26-0.84\frac{1}{{\left(F_{l}\right)}_{0}}\frac{{\left(C_{in}\right)}_{0}}{C_{d}}\frac{C_{d}}{C_{in}}\frac{{\left(P_{in}\right)}_{0}}{P_{in}}. \end{array}$$

**Figure 18:**
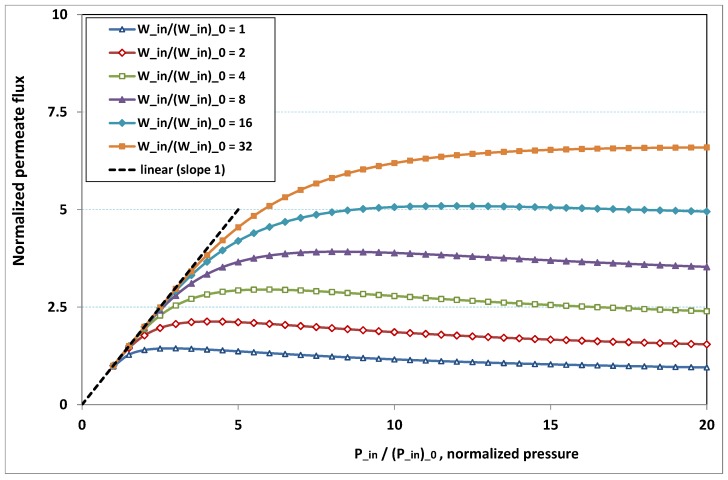
Fouling hindrance to permeation (1): normalized permeate flux $\varphi_{p}/{\left(\varphi_{p}\right)}_{0}$ vs. normalized pressure $P_{in}/{\left(P_{in}\right)}_{0}$ for various normalized feed flow $W_{in}/{\left(W_{in}\right)}_{0}$, with normalized feed concentration $C_{in}/{\left(C_{in}\right)}_{0}$ fixed to 1 (osmotic effects and pressure drop are supposed weak).

**Figure 19:**
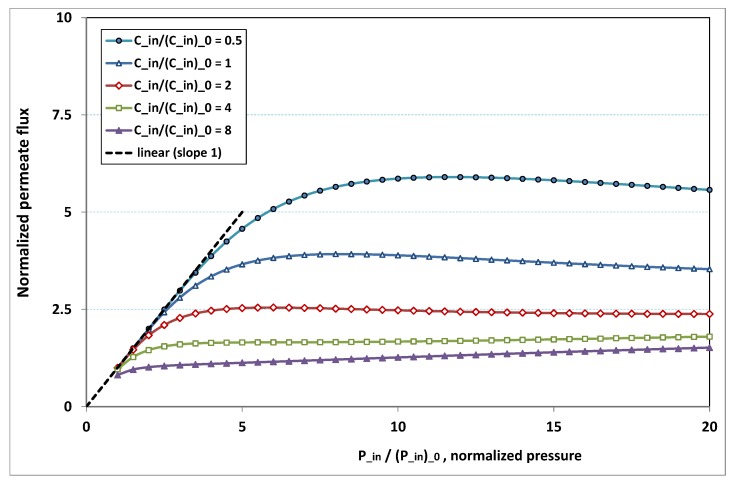
Fouling hindrance to permeation (2): normalized permeate flux $\varphi_{p}/{\left(\varphi_{p}\right)}_{0}$ vs. normalized pressure $P_{in}/{\left(P_{in}\right)}_{0}$ for various normalized feed concentration $C_{in}/{\left(C_{in}\right)}_{0}$, with normalized feed flow $W_{in}/{\left(W_{in}\right)}_{0}$ fixed to 8 (osmotic effects and pressure drop are supposed to be weak).

The plot of $\varphi_{p}/{\left(\varphi_{p}\right)}_{0}$, the normalized permeation flux, against $P_{in}/{\left(P_{in}\right)}_{0}$, the normalized pressure, is drawn in Figure 18 for various normalized feed flow $W_{in}/{\left(W_{in}\right)}_{0}$, with the normalized concentration $C_{in}/{\left(C_{in}\right)}_{0}$ being set to 1. On the other side, the same quantities are again plotted in Figure 19, but, for various normalized feed normalized concentration $C_{in}/{\left(C_{in}\right)}_{0}$, while the normalized feed flow $W_{in}/{\left(W_{in}\right)}_{0}$ is now set to 8. Note that the data of the curve for $W_{in}/{\left(W_{in}\right)}_{0}=8$ in Figure 18 are the same as the data of the curve for $C_{in}/{\left(C_{in}\right)}_{0}=1$ in Figure 19.

Both Figure 18 and Figure 19 present the same trends as the results reported in the experimental literature quoted in Section 1:

- at low pressure, no hindrance due to fouling occurs; the increase in permeation remains in direct ratio to pressure.
- the absence of fouling is maintained for higher pressures as feed flow (“the shear”) increases (Figure 18), or as feed concentration diminishes (Figure 19).
- when fouling arises, the dependence on pressure exhibits a saturation, and even a weakly marked maximum either for fixed feed flow (Figure 18), or for fixed feed concentration (Figure 19).
- when fouling takes place, this maximum increases either as the feed flow rate is enhanced (Figure 18) or as the feed concentration diminishes (Figure 19).

In both Figure 18 and Figure 19, the non-monotonous dependence of permeation on pressure is striking. This behaviour has already been observed in numerous experiments. What follows is an attempt at interpreting the occurrence of such a maximum in permeation as pressure increases. In Figure 19, a careful inspection shows that all the maxima happen for pressures that give a more or less constant value for $F_{l}$, say $F_{l}\approx 2$. Now, if we look at Figure 9, we observe that the whole channel is already affected by a serious fouling. Increasing pressure beyond these optimal pressures will impose fouling everywhere in the channel, with the subsequent deposit resistance, which seemingly increases faster than operating pressure. This can result from the fact that, when high polarization (and subsequent fouling) is achieved at the channel very entrance, the boundary layer is enlarged everywhere downstream, so that solute back-diffusion towards the bulk is lowered in the whole channel, then increasing concentration polarization, and therefore reducing permeation.

**Figure 20:**
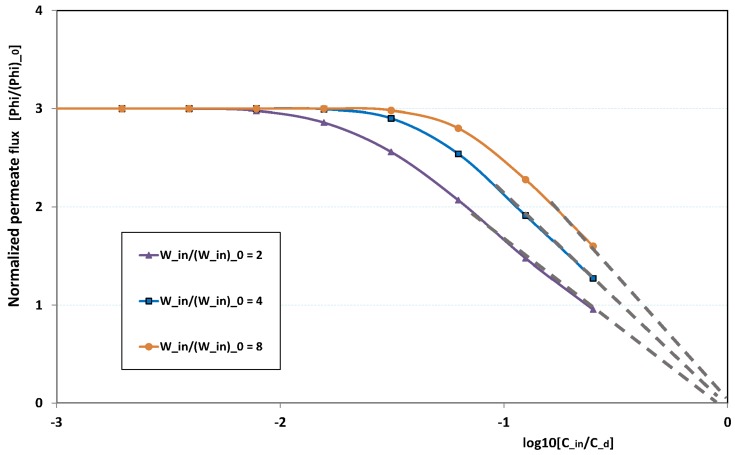
Fouling hindrance to permeation (3): normalized permeate flux $\varphi_{p}/{\left(\varphi_{p}\right)}_{0}$ vs. normalized solute concentration $C_{in}/C_{d}$ for various feed flow $W_{in}/{\left(W_{in}\right)}_{0}$ and with normalized pressure $P_{in}/{\left(P_{in}\right)}_{0}$ fixed to 3 (osmotic effects and pressure drop are supposed weak).

Figure 20 illustrates the role played by the inlet feed concentration for a given pressure. The figure plots the reduced permeation against $ln(C_{in}/C_{d})$ for three inlet flow rates. At low $C_{in}$, fouling is non-existent. This is why there is no variation with $C_{in}$, the permeation being determined by the trans-membrane pressure, which is here fixed to three times the pressure of reference. This is why the “clear water” permeation is three times the permeation of reference.

At larger $C_{in}$, fouling can arise, depending on the flow rate: at low flow rate, fouling starts for lower feed concentration than for high flow rate. When the feed concentration is high, permeation rapidly vanishes and tends towards zero linearly with $ln(C_{in}/C_{d})$, as observed in numerous experiments. As a result, all permeation curves seem to converge to nil for $C_{in}=C_{d}$, the critical concentration for deposit. Note these curves are not plotted for very high $C_{in}$, since our Prandtl model becomes inaccurate when $C_{in}$ is of the same order as $C_{d}$.

## 8. Conclusions

A situation of permeation controlled by Darcy’s law in cross-flow filtration has been studied together with the implications of a standard model of reversible fouling, which consists of imposing a material deposit, as long as a critical concentration is reached. Deposit stops—and the related wall resistance reaches a steady state—when the permeation diminishes and causes a polarization of concentration that corresponds to the critical concentration. The present contribution on reversible fouling can be summarized in several stages.

First, the general set of control parameters contains seven numbers: two numbers that control osmotic pressure and pressure drop have been chosen in such a manner that the related phenomena are minimized. As for the membrane number β, it is a vanishing quantity that justifies the use of the Prandtl approximation, which has then been studied numerically. The results have shown that mass transfer utterly controls filtration: this led us to discard the role played by $R_{in}$, the transverse Reynolds number. Hence, the intensive numerical study consisted in varying $Pe_{in}$, the Péclet transverse number, $N_{d}$, the deposit number, and λ, the reduced channel length. Furthermore, the analysis of the local permeation led us to interpret the results with the use of $F_{l}=Pe_{in}/N_{d}$, the so-called fouling number. Even though some scatter can be observed in this classification, the *z*-integration, which provides us with the channel overall permeation, markedly improves its quality.

It then turned out that all the numerical results can be gathered accordingly with two parameters: the channel reduced length λ and the fouling number $F_{l}=Pe_{in}/N_{d}$. This allowed us to search for a family of curves with a single parameter to perform the step of fit. This led us to the analytical expression (42), which provided us with the overall permeate flux in dimensional form as a function of all the parameters in dimensional form. Note that a crude use of expression (42) for $P_{in}\geq 40$ would neglect axial diffusion and therefore underestimate permeation.

Figure 18, Figure 19 and Figure 20 help us to appreciate the quality of the predictions brought by expression (42). They appear satisfactory, since they qualitatively exhibit all the features related to fouling experiments in ultrafiltration. In particular, the existence of a “critical flux” and of a “limiting flux” has been pointed out, as well as some non-monotonic dependence of permeation vs. pressure. Furthermore, numerous experimental configurations correspond to situations where mass transfer is mainly turbulent, owing to the resort to obstacles or vortices imposed on the feed flow. A straightforward manner to extend the present laminar approach to turbulence consists of introducing a rough modelling of turbulent transfer thanks to the turbulent diffusion coefficient $D_{turb}$ and in substituting it for $D_{0}$ in all the present analytical expressions.

Other regimes of filtration, such as reverse osmosis or nanofiltration, also experience fouling. Their interpretation can resort to the results of the present paper as a milestone. Strictly speaking, this needs to extend the present results to non-vanishing values of $N^{osm}$, which will conduct a rather complex discussion with four parameters. Nonetheless, the present approach confirms that the limiting flux phenomenon is intrinsic to fouling, to be compared with the logarithmic growth due to osmotic effects. In other words, the present study combined with Ref. [1] should help to discriminate both hindrance phenomena. 

## Nomenclature

$C_{in}$	inlet feed concentration (mol × m${}^{-3}$)
$C_{d}$	critical concentration for deposition (mol × m${}^{-3}$)
$N_{d}$	deposit number ($N_{d}\equiv C_{d}C_{in}^{-1}$)
*C*	dimensionless concentration (in units of $C_{in}$)
$P_{in}$	transmembrane pressure at inlet (TMP) (Pa)
$I_{0}$	membrane resistance to pure solvent filtration (kg × m${}^{-2}\times$ s${}^{-1}$)
$U_{in}$	filtration velocity for pure solvent feeding ($U_{in}\equiv P_{in}I_{0}^{-1}$) (m × s${}^{-1}$)
$\rho_{0}$	fluid density (uniform) (kg × m${}^{-3}$)
$\mu_{0}$	dynamical viscosity of the fluid (uniform) (kg × m${}^{-1}\times$ s${}^{-1}$)
*d*	channel half-spacing (m)
$R_{in}$	transverse Reynolds number of pure solvent filtration [$R_{in}\equiv \left(P_{in}\rho_{0}d\right){\left(\mu_{0}I_{0}\right)}^{-1}$]
$Pe_{in}$	tranverse Péclet number of pure solvent filtration [$Pe_{in}\equiv \left(P_{in}d\right){\left(D_{0}I_{0}\right)}^{-1}$]
Sc	Schmidt number of the feed [$Sc\equiv \mu_{0}{\left(D_{0}\rho_{0}\right)}^{-1}$]
$D_{0}$	solute diffusivity in the feed (uniform) (m${}^{2}\times$ s${}^{-1}$)
$W_{in}$	axial mean velocity at inlet (m × s${}^{-1}$)
$L_{de}$	“clear water” exhaustion length ($L_{de}\equiv I_{0}P_{in}^{-1}W_{in}d$) (m)
*L*	channel length (m)
λ	dimensionless channel length (in units of $L_{de}$)
$\Delta P_{HP}$	pressure drop along the channel (Pa)
α	Regirer number, $\alpha \equiv W_{in}P_{in}^{-1}\sqrt{\mu_{0}I_{0}d^{-1}}$
$P_{in}^{osm}$	osmotic pressure in the absence of polarization ($P_{in}^{osm}\equiv iRTC_{in}$) (Pa)
*i*	number of dissociated entities per molecule of solute
*T*	temperature of the solution (K)
*R*	perfect gas constant (J × K${}^{-1}\times$ mol${}^{-1}$)
$N^{osm}$	osmotic number, defined as $N^{osm}\equiv P_{in}^{osm}P_{in}^{-1}=iRTC_{in}P_{in}^{-1}$
$F_{l}$	fouling number, defined as $F_{l}\equiv Pe_{in}N_{d}^{-1}=\left(P_{in}C_{in}d\right){\left(I_{0}D_{0}C_{d}\right)}^{-1}$
$I_{f}\left(z\right)$	local resistance to permeation due to fouling (kg × m${}^{-2}\times$ s${}^{-1}$)
$R_{ec}$	recovery factor
$R_{f}$	fouling rate
